# Initiation of hepatitis C treatment in two rural Rwandan districts: a mobile clinic approach

**DOI:** 10.1186/s12879-021-05920-3

**Published:** 2021-02-25

**Authors:** Innocent Kamali, Dale A. Barnhart, Françoise Nyirahabihirwe, Jean de la Paix Gakuru, Mariam Uwase, Esdras Nizeyumuremyi, Stephen Walker, Christian Mazimpaka, Jean de Dieu Gatete, Jean Damascene Makuza, Janvier Serumondo, Fredrick Kateera, Jean d’Amour Ndahimana

**Affiliations:** 1 Partners In Health/Inshuti Mu Buzima, Rwinkwavu, Rwanda; 2 Department of Global Health and Social Medicine, Harvard Medical School, Boston, USA; 3 GlaxoSmithKline, Philadelphia, PA USA; 4 Rwanda Biomedical Centre, IHDPC, Kigali, Rwanda; 5 School of Population and Public Health, University of British Columbia, Vancouver, Canada

**Keywords:** Hepatitis C, Mobile clinic, Rural health, Rwanda

## Abstract

**Background:**

To eliminate hepatitis C, Rwanda is conducting national mass screenings and providing to people with chronic hepatitis C free access to Direct Acting Antivirals (DAAs). Until 2020, prescribers trained and authorized to initiate DAA treatment were based at district hospitals, and access to DAAs remains expensive and geographically difficult for rural patients. We implemented a mobile clinic to provide DAA treatment initiation at primary-level health facilities among people with chronic hepatitis C identified through mass screening campaigns in rural Kirehe and Kayonza districts.

**Methods:**

The mobile clinic team was composed of one clinician authorized to manage hepatitis, one lab technician, and one driver. Eligible patients received same-day clinical consultations, counselling, laboratory tests and DAA initiation. Using clinical databases, registers, and program records, we compared the number of patients who initiated DAA treatment before and during the mobile clinic campaign. We assessed linkage to care during the mobile clinical campaign and assessed predictors of linkage to care. We also estimated the cost per patient of providing mobile services and the reduction in out-of-pocket costs associated with accessing DAA treatment through the mobile clinic rather than the standard of care.

**Results:**

Prior to the mobile clinic, only 408 patients in Kirehe and Kayonza had been initiated on DAAs over a 25-month period. Between November 2019 and January 2020, out of 661 eligible patients with hepatitis C, 429 (64.9%) were linked to care through the mobile clinic. Having a telephone number and complete address recorded at screening were strongly associated with linkage to care. The cost per patient of the mobile clinic program was 29.36 USD, excluding government-provided DAAs. Providing patients with same-day laboratory tests and clinical consultation at primary-level health facilities reduced out-of-pocket expenses by 9.88 USD.

**Conclusion:**

The mobile clinic was a feasible strategy for providing rapid treatment initiation among people chronically infected by hepatitis C, identified through a mass screening campaign. Compared to the standard of care, mobile clinics reached more patients in a much shorter time. This low-cost strategy also reduced out-of-pocket expenditures among patients. However, long-term, sustainable care would require decentralization to the primary health-centre level.

## Introduction

Chronic hepatitis C infection affects 71 million people globally [1]. Untreated chronic hepatitis C can lead to cirrhosis of the liver, liver failure, and hepatocellular carcinoma, making hepatitis C one of the leading causes of liver cirrhosis and deaths [2]. Although Direct Acting Antiretroviral (DAA) treatment has been shown to cure over 90% of chronic hepatitis C cases [3–6] only 14 million people infected with hepatitis C virus know their status and only 1.1 million have initiated treatment [1]. In this context, the World Health Organization (WHO) has developed a campaign to eliminate hepatitis C by 2030 [7].

In sub-Saharan Africa, seroprevalence of hepatitis C is estimated at around 3% [8], which is approximately three times higher than estimates from general populations in Europe (0.54–1.5%) [9] and the United States (US) (0.9%) [10]. Rwanda, where hepatitis C prevalence estimates range between 6.8 and 9% among people over 25 years old and above, [11, 12] is the first country in sub-Saharan Africa to launch a national hepatitis C elimination plan. This ambitious plan exceeds WHO targets [7] and aims to screen 4 million people and treat at least 90% of identified cases by 2024 [13]. In response to this call to action, the Rwandan government has expanded access to rapid diagnostic testing (RDT) through national mass screening campaigns and has guaranteed free access to DAA medication for all people with chronic hepatitis. However, ensuring adequate linkage to care and treatment requires the decentralization of clinicians who are trained in hepatitis management. When Rwanda introduced the first national hepatitis prevention and management program in 2015, only 3 pharmacies in Rwanda were authorized to dispense drugs for hepatitis, all located in Kigali city [14].

By June 2018 the Rwandan government had trained infectious diseases clinical mentors, which included both medical doctors and nurses from district hospitals, and lab technicians to provide hepatitis management services at all district hospitals around the country [15]. Per national guidelines, both doctors and nurses are able to manage hepatitis B and C including clinical consultations, request for lab exams, treatment prescription, drug distribution and follow-up. However, accessing hepatitis C treatment can still be difficult and expensive for rural Rwandans. In addition to expenses related to traveling to and from the district hospitals, patients must also pay out-of-pocket for liver function and hematology laboratory tests, which are needed before DAAs initiation. Typically, laboratory tests and treatment initiation consultations occur on different days, increasing the transportation costs for patients. In order to reduce expenses and travel-related barriers to treatment initiation**,** we developed a mobile hepatitis clinic to improve access to care for patients with hepatitis C in Rwanda’s Kirehe and Kayonza districts. This paper describes the mobile clinic model and implementation experience, coverage, and costs associated with running the hepatitis C mobile clinic.

## Methods

### Setting

Kayonza and Kirehe are located in Rwanda’s eastern province, and are two of the three districts supported by Partners In Health/Inshuti Mu Buzima (PIH/IMB), a non-government organization that has been supporting Rwandan Ministry of Health since 2005 in health system strengthening. PIH/IMB supports Rwinkwavu and Kirehe district hospitals together with their 25 affiliated health centres, eight from Rwinkwavu and seventeen from Kirehe. On average, health centres are about 24 km from the hospital, which is over 8 h round trip on foot. Kayonza and Kirehe districts have hepatitis C antibody (Ab) positivity rates of 7.7 and 11.6%, respectively [16]. Mass screening campaigns were conducted in Kirehe and Kayonza in September 2019. Any patients testing positive for hepatitis C antibodies using a rapid diagnostic test and capillary blood were provided with a confirmatory Ribonucleic Acid (RNA) test using a venous blood sample and all patients with a detectable viral load (> 15 IU/L) were eligible for DAA treatment initiation.

### Description of the intervention

We developed the mobile hepatitis clinic to provide patients with access to DAAs treatment at primary-level health facility. Using a list of patients identified as eligible for hepatitis C treatment during the previous mass screening campaigns, we made a schedule to visit each health centre within the target districts. Patients who had screened positive for hepatitis B were also linked to care during the mobile clinic, but these patients reflected a minority of the patients linked to care were not included in this analysis unless they were co-infected with hepatitis C. Prior to the day of the mobile clinic visit, health centre staff contacted patients via telephone to schedule appointments. When patients could not be reached via telephone, we used the existing network of village-level community health workers to reach patients through home visits and inform the patient of the mobile clinic date. We also contacted each district pharmacy to ensure that a twelve-week course of DAA treatment could be reserved for all eligible patients to avoid any possible stock-outs. Clinicians were able to prescribe and dispense DAAs directly to the patients. On the mobile clinic day, we deployed a multidisciplinary team composed of (i) one physician or a nurse authorized by the Ministry of Health for hepatitis management, (ii) one laboratory technician, and (iii) a driver. We also provided two mobile laboratory machines, a Humalyser 3500 for biochemistry and Sysmex XP300 for hematology. We provided all necessary reagents to complete the pre-treatment initiation tests on the mobile machines, including Alanine Aminotransaminase (ALT), Aspartate Aminotransferase (AST), cell pack, and Stromatolyzer-WH; as well as other necessary medical supplies and commodities. Although the clinical team and laboratory machines were mobile, all clinical activities took place in the existing health centre infrastructure. All lab tests performed at the mobile clinic used venous blood samples.

At the mobile clinic, we assessed patient attendance and the clinician provided pre-treatment counselling in a group setting. Next, patients were sent to the laboratory technician for liver function and hematology tests. To minimize turn-around time, we made efforts to perform biochemistry and hematology tests in a single batch each day for all patients for a maximum of one hour and half between sample collection and result availability. Clinicians performed medical examinations that included physical consultations; nutritional status assessment through anthropometric measurements; evaluation of hepatitis C risk factors and patient family history; and assessment of extrahepatic manifestations such as skin rash, vasculitis, and co-morbidities. Clinicians used the liver function and hematology test results to calculate an Aspartate Aminotransferase to Platelet Ratio Index (APRI) score [17]. Patients with an APRI< 2 and no other clinical signs of decompensation received immediate treatment initiation. Patients with APRI score > 2 or other clinical signs of decompensation were referred to a specialist for liver ultrasound and hepatocellular carcinoma screening, per the national guidelines. Pregnant and breastfeeding women deferred treatment initiation until after they completed breastfeeding. Socio-demographic and clinical information were recorded in a paper-based patient file printed by the national hepatitis program. Overall, patients spent four to five hours at the mobile clinic to access all services. To facilitate patient follow-up, data from the hepatitis mass screening campaign and paper-based patient files were entered into a dedicated REDCap database used by PIH/IMB to manage hepatitis patients [18]. This study was approved by the Inshuti Mu Buzima Research Committee (IMBRC) and Rwanda National Ethics Committee (RNEC) 015/RNEC/2020) and all methods were performed in accordance with local guidelines and regulations. Because this study used retrospective data which was collected as part of routine clinical practice, informed consent was not obtained.

### Data analysis

We identified the number of people with chronic hepatitis C who had initiated on DAAs prior to the mobile clinic using data extracted from patient registries located at Kirehe and Rwinkwavu district hospitals. We assumed any patient who initiated hepatitis C treatment in Kirehe between November 18th, 2019 and January 31st, 2020 or in Kayonza between December 12th, 2019 and January 31st 2020, was a beneficiary of the mobile clinic campaign. To estimate coverage of the mobile clinic campaign, we identified patients who were eligible for treatment initiation through the mobile clinic using data extracted from the REDCap database. We defined patients as eligible for treatment initiation through the mobile model if they were: a) living in the catchment area of a PIH/IMB-supported health facility in Kayonza or Kirehe district, b) either screened positive for hepatitis C before January 31st, 2020 or had viral load test results indicating a detectable viral load for hepatitis C dated prior to January 31st, 2020, and c) had not started treatment prior to the start of the mobile clinic campaign in each district.

We used demographic data collected during the mass screening campaigns to compare characteristics of eligible patients who were and who were not able to be linked to care through the mobile clinic campaign using a Pearson’s chi-squared test. We reported risk ratios and 95% confidence intervals comparing the probability of being reached by the mobile clinic among patients with and without telephone number information and among patients with and without complete information on their address, defined as having information listed on the district, sector, cell, and village. This contact information was assessed for completeness, not for accuracy. All statistical analyses were conducted using Stata v.15.1 (Stata Corp, College Station, TX, USA).

To estimate the cost per patient of delivering mobile clinic services, we used an ingredients-based approach and a healthcare provider perspective. Following recommendations from the WHO Guide to Cost-Effectiveness Analysis [19], we included the costs of items typically covered by the PIH/ IMB operating budget in our analysis to reflect the opportunity cost of using these resources for the hepatitis C mobile clinic rather than for other activities. We categorized costs as either overhead, mobile clinic staff, capital, supplies and fees, and medication. To estimate overhead costs, we identified PIH/IMB permanent staff who were involved in organizing the campaign and allocated the value of their gross annual salary proportionally to the number of days they dedicated to the campaign. To reflect the cost of mobile clinic staff, we multiplied the daily salaries for the temporary laboratory technician and clinicians contracted to participate in the campaign by the number of days of the campaign. To estimate the cost of the driver, we allocated the driver’s gross annual salary proportionally to the number of days dedicated to the mobile clinic campaign. The cost of capital, including vehicles and laboratory machines, was estimated by calculating the annualized purchase price of these items over the anticipated lifetime of the item assuming an annual discount rate of 10%. To calculate the daily cost of these items, we divided the annual cost by 260 to reflect the number of working days in a year. The cost of supplies and fees were identified from the mobile clinic’s operating budget. We estimated the cost of DAA medication based on the government of Rwanda’s negotiated price with the drug manufacturer [20]. We reported the cost-per-patient of our program both with and without the cost of DAAs. In Rwanda, these are provided by the government free of charge, and providing mobile clinic services to patients does not include the price of these drugs. However, we recognize that in other settings the mobile clinic team may also be responsible for covering the cost of drugs. After identifying the total cost of running the mobile clinic, we divided the cost by the total number of patients with hepatitis C initiated to estimate cost per patient. All costs were converted from Rwandan francs to U.S. dollars using an exchange rate of 920 francs per dollar. To estimate the reduction in patients’ out-of-pocket expenses associated with initiating treatment through the mobile clinic rather than through the standard of care, we assumed that the major differences in out-of-pocket expenses would be the cost of transport from the local health centre to a district hospital and the cost of covering pre-treatment laboratory tests, which are not currently covered by Rwanda’s Community Based Health Insurance program (CBHI/Mutuelle). We used the cost of lab tests under CBHI/Mutuelle in our primary analysis because it is the most affordable and widely subscribed to insurance program and covers 81.6% of Rwandans [21]. However, we also report the estimated reduction in out-of-pocket costs among the uninsured. We did not include the cost of transport from the home to the local health centre because most patients travel to health centres on foot and because this cost would be the same both before and after the initiation of the mobile clinic. We estimated an average round trip cost of transportation from health facilities to district hospitals in Kayonza and Kirehe districts using standard PIH/IMB transportation reimbursement levels. We used the current prices of medical services offered at district hospitals to estimate the out-of-pocket costs for necessary laboratory tests under CBHI/Mutuelle and among the uninsured [22]. Costing analyses were conducted using Microsoft Excel.

## Results

### Number of patients initiated before and during the mobile clinic

**Fig. 1:**
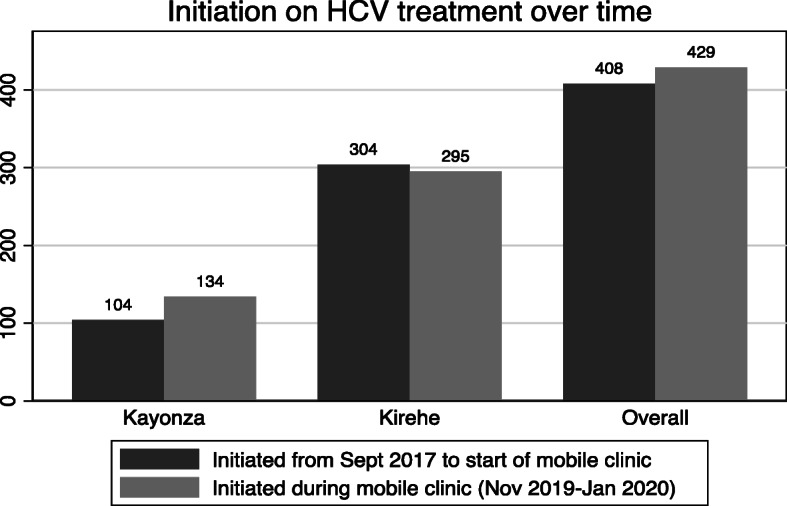
Comparison of patients with hepatitis C initiated before and during mobile clinic

Between the availability of hepatitis C treatment program in Kirehe and Rwinkwavu district hospitals in September 2017 and the start of the mobile clinic program, 408 patients initiated treatment for hepatitis C. Two patients from Kayonza and 13 from Kirehe exhibited APRI score > 2 or other clinical signs of decompensation were referred to a specialist for liver ultrasound and hepatocellular carcinoma screening, per the national guidelines. Overall, the 429 of patients initiated during eleven-week period of mobile clinic campaign exceeded the total number of patients initiated during the previous 25 months of the hepatitis treatment program (Fig. 1).

### Coverage of mobile clinic campaign

Using the REDCap database, we identified 661 patients with chronic hepatitis C in Southern Kayonza and Kirehe districts who were eligible for hepatitis C treatment initiation during the mobile clinic campaign. The 429 patients who were linked to care reflect program coverage of 64.9% (95% CI: 61.1–68.5%). Mobile clinic coverage was 71.3% in Southern Kayonza and 62.4% in Kirehe (Table 1). There was no difference in coverage between males and females, age, socioeconomic status, insurance status, marital status, or profession.

**Table 1:** Characteristics of patients. The overall samples size was 661. Sample sizes are given for each variable to reflect missing data

Factor	Not reached by mobile clinic	Reached by mobile clinic	*p*-value
	*N* = 232	*N* = 429	
PIH Supported Site			0.030
Kayonza	54 (28.7%)	134 (71.3%)	
Kirehe	178 (37.6%)	295 (62.4%)	
Sex (*N* = 655)			0.77
Female	160 (35.5%)	291 (64.5%)	
Male	70 (34.3%)	134 (65.7%)	
Age (years), median IQR	60 (48–72)	62 (50.5–70)	0.46
Ubudehe (*N* = 474)			0.16
Category 1	25 (26.3%)	70 (73.7%)	
Category 2	29 (17.2%)	140 (82.8%)	
Category 3	50 (24.4%)	155 (75.6%)	
Not known	0 (0.0%)	5 (100.0%)	
Insurance Status (*N* = 501)			0.54
No insurance	0 (0.0%)	1 (100.0%)	
CBHI/Mutuelle	74 (15.1%)	416 (84.9%)	
RSSB	0 (0.0%)	9 (100.0%)	
Other	0 (0.0%)	1 (100.0%)	
Marital Status (*N* = 492)			0.74
Single	8 (21.1%)	30 (78.9%)	
Married or Cohabitating	40 (14.6%)	234 (85.4%)	
Widowed	25 (14.6%)	146 (85.4%)	
Divorced	1 (11.1%)	8 (88.9%)	
Profession category (*N* = 449)			0.22
Farmer	59 (14.3%)	353 (85.7%)	
Unskilled Labor	2 (13.3%)	13 (86.7%)	
Skilled Labor	0 (0.0%)	2 (100.0%)	
Professional	0 (0.0%)	12 (100.0%)	
Student	3 (37.5%)	5 (62.5%)	
Telephone recorded at screening (N = 661)			< 0.001
No	123 (57.7%)	90 (42.3%)	
Yes	109 (24.3%)	339 (75.7%)	
Full address collected at screening (*N* = 661)			< 0.001
No	108 (75.5%)	35 (24.5%)	
Yes	124 (23.9%)	394 (76.1%)	
Screened during September 2019			< 0.001
Yes	59 (14.2%)	357 (85.8%)	
No	173 (70.6%)	72 (29.4%)	

Patients with their full address recorded during screening were 3.10 times more likely to be reached during the mobile clinic (95% CI: 2.32, 4.16) while patients who had a telephone number recorded at screening were 1.79 times more likely to be reached during the mobile clinic (95% CI: 1.51–2.11). In general, missing data was much less common among the 416 patients identified during the September 2019 mass screening campaigns than among the 245 patients identified in previous campaigns. Data from these previous campaigns exhibited 61.2% missingness for telephone number, 40.8% missingness for complete address, and over 50% missingness for ubudehe, insurance status, marital status, and profession.

#### Cost of running the mobile clinics

As shown in Table 2, the total cost per patient initiated on hepatitis C treatment, including the cost of DAAs, was $89.36. DAAs reflected 67% of the total program cost while mobile clinic outreach activities cost $29.36 per person, reflecting only 32.8% of the total cost of the program. Besides DAAs, the largest costs associated with mobile clinic implementation were overhead and transportation.

**Table 2:** Cost of hepatitis C mobile clinic from the health care provider perspective. All costs given in US dollars

			Total
**Administration and Overhead**^**1**^			5606.45
**Mobile Clinic Staff**	**Daily Salary**	**Days**	
Clinician	15.53	34	527.92
Lab Tech	15.53	34	527.92
Driver	13.53	34	460.06
**Capital**	**Annualized Daily Cost**	**Number of Units**	
Vehicles^2^	38.48	34	1308.32
Human Biochemistry 3500^3^	2.04	34	69.40
Sysmex XP 300 Hemotology^4^	6.12	34	208.19
**Fees, Supplies, and Materials**	**Cost per Unit**	**Number of Units**	
Per diem fees for PIH staff	2.18	102	222.36
Per diem fees for HC staff	15.53	68	1056.04
Daily fuel	11.96	34	406.64
Reagents			
ALAT	1.19	429	510.51
ASAT	1.19	429	510.51
Hematology	2.11	429	905.10
Other commodities			
Alcohol Swabs	0.33	429	139.89
Cotton	0.11	429	46.63
Gloves	0.13	429	55.96
Needles	0.07	429	28.91
Tubes	0.01	429	4.29
**Medication**	**Cost per Unit**	**Number of Units**	
DAAS	60.00	429	25,740.00
	***TOTAL COST OF PROGRAM***		**38,335.11**
	***TOTAL COST PER PATIENT***		**89.36**
	***TOTAL COST PER PATIENT, EXCLUDING DAAS***		**29.36**

^1^Overhead was calculated by allocating annual gross of salaries of ID team members proportionally to the total number of days each permanent ID staff member dedicated to this project

^2^Annualized cost of a vehicle assumes a purchase price of 76,087 an annual discount rate of 10 and a useful lifespan of 15 years

^3^ Annualized cost of Human Biochemistry 3500 machine assumes a purchase price of 3260 an annual discount rate of 10% and a useful lifespan of 10 years

^4^Annualized cost of Sysmex XP 300 Hemotology machine assumes a purchase price of 9782 an annual discount rate of 10% and a useful lifespan of 10 years

#### Out of pocket costs for patients

When estimating the expected out-of-pocket expenses among patients seeking to initiate DAA treatment, we found that patients covered using CBHI/Mutuelle program could expect to pay $ 9.87 more in out-of-pocket under the standard of care compared to under the mobile clinic model. For patients without CBHI/Mutuelle, the reduction in out-of-pocket expense was estimated at $19.71 (Table 3). This reduction is an underestimation of the true cost savings for the patients as it does not include the reduction in opportunity costs associated with decreasing the number of days patients spent in the clinic from two to one.

**Table 3:** Reduction in out of pocket expenses for mobile clinic patients (USD)

Item	Cost reduction with CBHI/Mutuelle	Cost reduction without CBHI/Mutuelle
Transport from health centre to hospital	$5.86	$5.86
ALT	$1.19	$4.11
AST	$1.19	$4.11
Hematology test	$1.63	$5.62
Total	**$9.87**	**$19.71**

## Discussion

Our experience demonstrates that mobile hepatitis clinics are a feasible treatment strategy to promote same-day treatment initiation for patients with hepatitis C in resource-constrained settings. Through our mobile clinics, we were able to initiate 64.9% of all patients awaiting treatment on DAAs during an eleven- week period. The number of patients initiated on treatment through the mobile clinic program exceeded the total number of patients who were initiated on treatment under the standard of care during the previous 25 months. While some of this difference may reflect expanded testing capacity in Rwanda as new initiatives that have been introduced, for example the adoption of rapid diagnostic tests and same-day venous blood collection for viral load testing during the mass screening campaigns, our results demonstrate that mobile clinics can be used to ensure prompt linkage to care where services are not decentralized. This strategy is especially useful following mass screening campaigns, when large numbers of patients are awaiting treatment initiation. To our knowledge, this is the first hepatitis C treatment mobile clinic program model implemented in Rwanda and sub-Saharan Africa. Our program may serve as a model elsewhere in Rwanda and to other countries seeking to scale up access to hepatitis C treatment.

When including the cost of the DAAs in our program, we found that the costs of implementing the mobile clinic system reflected only 32.8% of the total cost per patient initiated. In Rwanda, where the national government is committed to providing DAAs free-of-charge to all patients with hepatitis C, this relatively small increase in per-patient costs may be an important investment to ensure adequate linkage to care and equitable access to treatment for all citizens. Although we did not conduct a formal cost-effectiveness analysis, the per-patient cost of this program compares favorably with an antenatal care program in Rwanda where the first visit costs $21 per woman [23].

The cost of our program also compares favorably to the cost of HIV care and treatment visits and antiretroviral therapy, which is estimated to cost an average of $208 per patient per year in Rwanda, Ethiopia, Malawi and Zambia [24].

Mobile clinics have successfully increased access to care in rural settings for other programs, including prenatal care, HIV, and other sexually transmitted infections [25]. Mobile clinics can be used to both reduce patient costs and improve health outcomes in underserved and vulnerable populations [26]. Since 83% of Rwandans live in rural areas [27] many of them have to walk long distances or arrange costly transport to access health services at district hospitals. We estimated that our mobile clinic program was able to cut transport time in half and reduce patients’ out-of-pocket expenses by $9.87. This is a meaningful cost reduction in a country where 43.1% of the rural population live under poverty and 18.1% in an extreme poverty [22]. Over one third of the reduction in out-of-pocket costs is attributable to our provision of free liver and renal function tests through the mobile clinic program. To strengthen support for vulnerable patients and to promote the goals of the national hepatitis C elimination campaign, the government of Rwanda may consider including liver and renal function tests as a covered service in CBHI/Mutuelle package at health centre for patients with viral hepatitis.

During the implementation of the mobile clinic, not all expected patients could be reached to schedule their appointment. Although we worked with community healthcare workers to seek patients in their home villages, some patients were impossible to contact, possibly because information was entered incorrectly during the mass screening campaigns. As evidenced by our analysis, having complete data on telephone number and address were strongly associated with being able to be linked to care during the mobile clinic campaign. To improve the effectiveness of these mass-screening campaigns, we recommend the adoption of high-quality training for data collectors, investment in an electronic data capturing system, and real-time monitoring and feedback to ensure that data collectors are collecting information as accurately as possible. When we have been able to implement these strategies in subsequent campaigns, we have found that it has substantially improved our ability to link patients to care, as partially evidenced by the lower levels of missing data during the September 2019 campaign compared to previous campaigns. Importantly, we did not observe differences in mobile clinic coverage by age, gender, or socioeconomic status, suggesting coverage of the mobile clinic program was relatively equitable across demographic groups.

We identified several additional lessons learned while implementing the mobile clinic campaign. First, we were unable to provide ultrasound exams for patients with hepatitis who presented with suspected liver decompensation. Although there were very few cases with suspected liver decompensation, the availability of a mobile ultrasound machine could have improved our ability to provide same-day initiation for these patients. Second, clearly communicating with the patients the starting hour for the mobile clinic was critical for enabling collective pre-treatment counselling, processing biochemistry and hematology tests in one batch, and ensuring that teams had enough time to initiate all patient by the end of the day. Finally, our mobile clinics were implemented following a mass screening campaign that identified a large number of patients who required treatment initiation in a short period of time. However, the cost-effectiveness of this strategy depends on having a relatively large number of patients awaiting treatment per health centre, as would typically be the case after a mass screening campaign. Ultimately ensuring long-term, sustainable, decentralized access to hepatitis treatment requires task shifting, where health centre-level nurses are trained to manage hepatitis on a daily basis. Decentralization of care has been demonstrated model to be feasible by the HIV task-shifting model, where nurses who have been well trained, mentored, and given support can effectively manage HIV treatment [28, 29].

During our campaign, we did provide infectious disease nurses working at health centres with training on hepatitis management, and, in collaboration with the Ministry of Health, we have also been supporting theoretical and practical training sessions to allow these nurses to become certified in hepatitis management.

Our analysis has some limitations. We used clinical records to assess the number of patients initiated to treatment before and during the period of mobile clinic geographic and budgetary data to assess costs associated with the program. These data sources were not intended for research purposes and may suffer from missing or incomplete data. Furthermore, because medical records did not include explicit information about mobile clinic participation, we had to rely on dates of screening, viral load testing, and treatment initiation to assess mobility clinic eligibility and participation. However, because we were the only health care provider offering hepatitis treatment in our catchment area and because our results are very similar to daily records kept by mobile clinic staff during the campaign, we believe any misclassification is minimal. As part of our campaign, we also initiated some patients who had screened positive for hepatitis B; however, we did not include these patients in this analysis. This decision reflects the fact that, unlike hepatitis C, hepatitis B does not currently have a cure, requires life-long treatment, and is less suited to short-term campaigns than hepatitis C. Including patients with hepatitis B in our costing analysis would have reduced the per-patient costs of the overall campaign. Finally, we did not compare the quality of care provided during mobile clinics to what was provided prior to the initiation of the mobile clinic program. Despite these limitations, we believe this analysis demonstrates the feasibility of a mobile clinic-based model for hepatitis C treatment initiation, and hope that it can be used to inform future interventions in similar contexts.

## Conclusion

Access to hepatitis C treatment in Rwanda is improving but is still limited in rural settings. Implementing a mobile clinic program with basic laboratory services is a feasible and potentially scalable tool to increase access to treatment. This low-cost strategy can complement to mass screening campaigns by linking large numbers of patients to care in a short period of time. The model also reduces time spent at a health facility and out-of-pocket expenditures for patients. The model could potentially be extended to other settings or to other diseases requiring linkage to care for short-term curable diseases in rural settings.

## Data Availability

The datasets for this paper are not publicly available but are available from the corresponding author on reasonable request.

## Abbreviations

ALT: Alanine transaminase
AST: Aspartate transaminase
APRI: Aspartate Aminotransferase to Platelet Ratio Index
CBHI: Community Health Insurance
CI: Confidence Interval
DAAs: Direct Acting Antivirals
IHDPC: Institute of HIV Disease Prevention and Control
IMBRC: Inshuti Mu Buzima Research Committee
IQR: Interquartile range
PIH/IMB: Partners In Health/Inshuti Mu Buzima
MoH: Ministry of Health
RDT: Rapid Diagnostic Test
RBC: Rwanda Biomedical Centre
REDCap: Research Electronic Data Capture
RNA: Ribonucleic Acid
RNEC: Rwanda National Ethics Committee
USD: US dollar
WHO: World Health Organization
